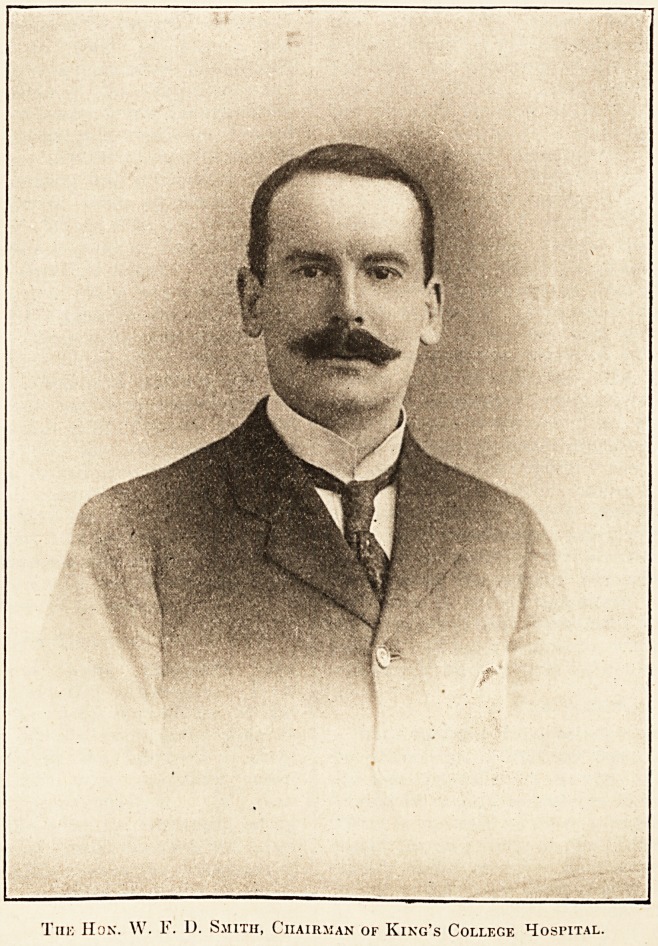# Eminent Chairman Series, VI. The Hon. W. F. D. Smith

**Published:** 1911-03-11

**Authors:** 


### SPECIAL INSTITUTIONAL ARTICLE.

### EMINENT CHAIRMAN SERIES.*

### VI.—THE HON. W. F. D. SMITH, Chairman of King's College Hospital.

The conditions with which those responsible for the management of King's College Hospital are confronted at the present time are probably unique. They have not only to do their best to maintain the undiminished usefulness and efficiency of a great general hospital in the heart of London, but at the same time they have to accomplish the erection in South London of a new general hospital which shall, when completed, be three times the size of the present King's College Hospital. In the very nature of things this double effort must be at once serious and long-sustained. Indeed, as a matter of fact, the decision to remove King's College Hospital from the West Central district to that part of South London which so sorely needs hospital service, was taken more than seven years ago, and since that time the work of preparation and building has been steadily going on. To-day, as the outcome of tremendous effort and wide appeal, a large portion of the new hospital has arisen on the slopes of Denmark Hill—not, of course, the whole building that has been planned, but still a large and important section, sufficient to show the excellence of the design and the difficulties with which the management has had to strive. So far advanced, indeed, are the buildings that the London Press has already had an opportunity of inspecting the beginnings of the several blocks, as recorded in our columns of February 25 last.

The Hon. W. F. D. Smith, Chairman of King's College Hospital.

The tremendous responsibilities which rest upon the chairman of an institution under conditions of such development and progress will be apparent to all hospital workers. Previous articles in this series have laid stress on the fact that the chairmen of our great voluntary charitable institutions are men who do not shrink from giving loyal personal service, to an extent which only those who are intimately acquainted with the demands made upon the time and energy of these gentlemen can adequately appreciate, in order to further the interests of the institutions with which they are connected. When to the normal work there is added the special burden which the unique circumstances connected with the removal of King's College Hospital to its new southern site involve, one can easily understand that the task of the institutional chairman assumes gigantic proportions, and that the responsibilities which he must feel are greatly increased. In such circumstances, even more than in others, the chairman is no mere figurehead—no mere occasional president of meetings. He must be constantly at hand and easily available for the settlement of innumerable questions that daily occur in the conduct of a great institution. Bearing all

* The previous articles in this series appeared in The Hospital of October 1, November 1, December 10, January 14, and February 11.

this in mind, it is no mere formality when we state that King's College Hospital is truly fortunate in its present chairman.

A Biographical Note.

The Hon. W. F. D. Smith is the well-known son of a well-known father, the late William Smith, M.P. He was born in the year 1868, and received his education at Eton and Oxford, distinguishing himself at both places by scholastic achievements and by his prowess in sport. On completing his University course, and after taking honours in the Modern History school, Mr. Smith proceeded on an extended tour of the world, visiting Canada, Japan, and India. Returning home in 1891, he almost immediately entered his father's firm—that of W. H. Smith and Son—as a junior partner, and threw himself whole-heartedly into the work, with the result that he has made himself thoroughly familiar with the various departments of that huge business. When, through the sudden death of his father in the autumn of 1891, great resposibilities were thrust upon him, he was therefore eminently qualified to take up the burden. To-day, in spite of his numerous outside activities, he is the head of the great firm that bears his name, and it would be difficult to find any business house in which the cordial relations existing between employer and employés are so strongly marked as in this one which he controls.

His Connection with King's.

In 1908, when he was a Member of Parliament, representing his father's old constituency, the Strand Division, Mr. Smith succeeded Lord Methuen as Chairman of King's College Hospital. He had always taken a keen and active interest in the working of the institution, and his firm has been closely identified with the hospital, college, and school. He gained sterling experience through serving on the Committee of Management, and has been prominently associated with the scheme for the removal of the hospital to a site outside the congested metropolitan area. The story of his work for the hospital is the story of the new institution now nearing completion on the slopes of Denmark Hill. In the columns of The Hospital the details of the scheme have been faithfully outlined year after year, but it is well to cast a glance back and trace the beginnings of the undertaking that has been so successfully carried out In May, 1902, at the special festival dinner, Sir John Cockburn outlined the important reasons for enlarging and improving the institution, and pointed out that it was imperative, in order to render the hospital modern in the best sense of the term, to expend a sum of at least £75,000 on reconstruction. Lord Dillon laid further stress on the difficulties of improving the buildings in Portugal Street, which were out of date and so hemmed round that it was possible only to extend skywards—a suggestion which the County Council would veto. The activities of the hospital had extended tremendously, and the strain on the 244 beds was becoming greater every year.

The Removal Scheme.

In such circumstances, after due consideration, the proposal to remove the hospital to a suburban site was adopted. A summary of what had been done up to 1906 was furnished in the admirably lucid speech by Lord Milner at the festival dinner in that year (The Hospital, June 2, 1906, pp. 165-6), in which he stated that "the great stride forward which the institution had made could only be realised when it was borne in mind that the new institution would have all the necessary arrangements to meet the highest scientific requirements of modern hospital progress." The site for the new hospital was a matter that demanded long and earnest consideration, but the Committee's difficulties were ended by the generous gift of a valuable plot of land in South London by Mr. Smith himself. This important point having been settled, no time was lost in proceeding with the scheme. The building committee decided to hold a limited competition, several well-known architects, with experience of hospital construction, were invited to send in plans, and Mr. Rowland Plumbe was appointed adjudicator. The special requirements of the Committee, and the fact that the site of twelve acres, though large, did not admit of an extravagant treatment, limited the architects to some extent in their choice of design, but the competition was productive of an interesting and instructive series of plans. The final award was to Mr. W. A. Pite, and our comment on the adjudication is given in The Hospital of January 4, 1908, where we stated: "The decision of the assessor was in every way a right one; Mr. Pite's plans showed a grasp of the subject and a knowledge of detail which were only approached by one other competitor, whose plans were placed second. No hospital building of anything like the importance of this one has been undertaken in London since the erection of St. Thomas' Hospital some forty years ago; and the completion of this great scheme will be looked forward to with the liveliest interest by all engaged in hospital work." Now that it is possible to see, in actual existence, the fine plan which Mr. Pite produced—or at least sufficient of it to form some idea of what the whole will be like when it is finally completed—the truth of that statement admits of no dispute.

### The New Hospital.

The new hospital costs £300,000, and has accommodation for 600 in-patients. It is placed in a position where it will be of infinitely more value to the community than is the present hospital, in the heart of a great and growing population which urgently needs increased hospital accommodation. It contains provision for the adequate treatment, along the best modern lines, of special diseases, and when completed it will be able to boast the possession of special departments unrivalled in point of excellence and completeness by any other London hospital. As an institution it will be able to claim that it is superior, so far as planning and construction on the most approved modern principles can make it, to any Metropolitan hospital, and to rank as one of the most beautiful of our voluntary institutions in this country.

### The Question of the Out-patient Department.

Such a result has not been brought about without much labour and care on the part of the Chairman and his colleagues. The appeal fund, for example, needed vigorous advertising and involved much personal service on the part of those who undertook the gigantic responsibility of soliciting aid from a public already over-appealed to. The splendid record of King's, the equally splendid generosity of its supporters, chief among whom was Mr. Smith himself, and the fact that the public, as a whole, cordially approved of the removal scheme, sufficed to make that appeal a success. The initial amount needed for the starting of building operations was subscribed, work was begun, and, as already stated, the hospital is now in a fair way of being able to treat out-patients in the course of the ensuing two years. A matter which reflects the greatest credit on all concerned in the undertaking has been the treatment of the professional objections to the scheme. Local medical opinion was at first strongly opposed to the introduction into the Camberwell district of a teaching hospital with a large out-patient department. The authorities of King's College Hospital, as we pointed out in a leading article dealing with the matter (The Hospital, May 9, 1908, p. 138), by appointing a watching committee showed that they were not indifferent to the legitimate interests of the private practitioners in the district where the new hospital was to be erected. This committee considered the question in all its bearings, and consulted with special committees appointed by the local practitioners in all friendliness. The local practitioners urged the adoption of several recommendations with regard to the treatment of out-patients; for a detailed account of their resolutions we must refer our readers to the reports which appeared in The Hospital of May 1908. Suffice it here to say that the authorities of King's approved of nearly all these recommendations, which were precisely along the lines advocated by this Journal for many years. By so doing the hospital authorities admitted that they were in perfect agreement with the principle that the out-patient department should be an auxiliary to, and not a competitor with, the local doctor, and should be used only for consultation purposes.

### Pay Wards.

With regard to the question of paying in-patients, the authorities maintained their attitude, although the local practitioners strongly disapproved of the admission of private patients to the general wards. There is no need to enter into this subject at this stage; our own views on the matter have been sufficiently stated on more than one occasion, and we feel sure that local practitioners, by this time, will be ready to reconsider their objections to such admission. The important fact on which stress should be laid is that by their action in meeting the local committees and adopting out-patient rules in consultation, the authorities of King's have gone far to remove the one great stumbling-block to the prevention of hospital abuse—the want of sympathy and co-operation existing between the hospital specialist and the local practitioner. The regulations drawn up for the out-patient department of the new institution on Denmark Hill—which provide for the investigation of every case that comes for treatment and for the adequate safeguarding of the interests of the doctor—are in every way excellent, and in framing them the hospital authorities have set an example which sooner or later will have to be followed by every hospital in this country.

### The Medical School.

On September 1, 1910, the King's College, London (Transfer) Act was passed, whereby the affairs of the hospital were entirely separated from those of the College. Under the provisions of this Act the Committee of Management has from that date been able to govern the hospital and control its affairs without any reference to the council of King's College. The other measure which affected the hospital was the King's College Hospital (Removal) Act, which entrusted the authorities with definite powers for transferring the institution to the new site, and with the management of the advanced medical school. This advanced school, as distinguished from the school in which the preliminary work is carried on, will be at the new hospital. Already King's College Medical School is by many considered to be the premier medical school in London, and without going so far as to endorse that opinion, we have no hesitation in saying that it will certainly have the finest opportunities for development in its new quarters.

With all these extensions of hospital activity during the last five years Mr. Smith has been prominently associated. "His energy is constantly apparent to all those who know the hospital and its working," writes one who has been actively associated with him in the work of reconstruction and the appeal for funds. "From the very first he set himself to fulfil the great tasks before him with energy, splendid liberality, and a serious sense of the responsibilities of his office as Chairman. No amount of sheer hard work and personal effort is begrudged the hospital by its Chairman, who is to be found day after day presiding, not merely at the ordinary hospital meetings, but at the various sub-committees concerned with the details of the new hospital and the working of the institution in Lincoln's Inn Fields. All who have to do with the finances of the hospital and of the appeal fund are aware of the great aid which he has given by means of his generous gifts which he has made and is now making to these funds. In the early days of the removal scheme he presented the magnificent site of twelve acres for the new hospital at Denmark Hill, at a cost of £66,000, and early in the present year he intimated to the Committee of Management that he would be responsible for the erection of one of the two central ward blocks of the new hospital—a gift which means the outlay of something like £30,000, and will give to the institution more than seventy new beds. These great and outstanding gifts by no means exhaust the Chairman's liberality to King's College Hospital, and to the other institutions associated with its name. At the present moment Mr. Smith is working hard to promote the special Coronation Year Appeal for the completion of the new hospital—an appeal, as he observed at the last dinner of the hospital, which has always been running but which has not been hard pushed during the last eighteen months. The time has now come to ask for sufficient funds to complete the splendid institution designed by Mr. Pite. A sum of not less that £150,000 is still needed, and with this it is hoped that part, at any rate, of the great scheme will speedily be accomplished and the opening of the new King's College Hospital in South London be brought within measurable distance." In this great project Mr. Smith has the good wishes of all who know him, and more especially of those who are privileged to be associated with him in this splendid endeavour!

## Figures and Tables

**Figure f1:**